# Structural Analysis of Calcium Phosphate-Based Submicrospheres with Internally-Crystallized Iron Oxide Nanoparticles Fabricated by a Laser-Assisted Precipitation Process

**DOI:** 10.3390/ma12244234

**Published:** 2019-12-17

**Authors:** Maki Nakamura, Ayako Oyane

**Affiliations:** Nanomaterials Research Institute, National Institute of Advanced Industrial Science and Technology (AIST), Central 5, 1-1-1 Higashi, Tsukuba, Ibaraki 305-8565, Japan; a-oyane@aist.go.jp

**Keywords:** calcium phosphate, iron oxide nanoparticles, cross-sectional analysis, submicrospheres, laser-assisted precipitation process

## Abstract

Calcium phosphate (CaP)-based submicrospheres containing magnetic iron oxide (IO) nanoparticles (IO–CaP submicrospheres) have potential for various biomedical applications. We recently achieved facile one-pot fabrication of IO–CaP submicrospheres using a laser-assisted precipitation process in which weak pulsed laser irradiation was applied to a labile CaP reaction mixture supplemented with ferrous ions under adequate pH. In this study, we performed cross-sectional transmission electron microscopy (TEM) analysis of the resulting IO–CaP submicrospheres. The cross-sectional TEM analysis revealed that the IO–CaP submicrospheres were heterogeneous in their internal nanostructures and could be categorized into two types, namely types A and B. The type A submicrospheres contained single nano-sized IO nanoparticles homogeneously dispersed throughout the CaP-based matrix. The type B submicrospheres contained larger IO nanoparticles with an irregular or spherical shape, which were mostly a few tens of nanometers in size along with one or two submicron-sized domains. These findings provide new insight into the formation mechanism of IO–CaP submicrospheres in this fabrication technique as well as future applications of the resulting IO–CaP submicrospheres.

## 1. Introduction

Magnetic iron oxide (IO) nanoparticles have considerable potential for various biomedical applications such as heating elements in hyperthermia, transfer agents in magnetofection, and magnetic resonance imaging (MRI) contrast agents [1,2,3]. In these biomedical applications, the structure of magnetic IO nanoparticles is considerably important. For example, the MRI relaxivity of magnetic IO nanoparticles varies with their structures such as particle geometry (size, shape), crystal structure, surface chemistry, and aggregation state [4,5]. Additionally, the heat dissipation mechanism (e.g., hysteresis loss, Néel relaxation, and Brown relaxation) and the heating efficiency of magnetic IO nanoparticles in hyperthermia largely depend on the particle sizes [4,6]. Therefore, preparing magnetic IO nanoparticles with an appropriate structure for each application purpose is important.

To deliver IO nanoparticles into the body, nano- and submicron-sized calcium phosphates (CaP) particles loaded with IO nanoparticles have been developed [7,8,9]. These CaP particles are able to carry a large number of IO nanoparticles; hence are expected to deliver IO nanoparticles to the target site efficiently when having a suitable surface and geometry. CaPs are employed as a carrier matrix of IO nanoparticles, because they show low toxicity and good biocompatibility owing to their similarities to the mineral components found in human bones and teeth [10,11,12,13]. Conventionally, these IO-loaded CaP particles have been fabricated by time-consuming and/or multistep chemical precipitation processes [14,15,16,17]. For example, Mondal et al. reported a two-step process in which IO nanoparticles were prepared and subsequently treated with CaP solutions to produce IO-loaded CaP particles [15]. Ansar et al. reported one-pot fabrication of IO-loaded CaP particles, however, their processing time was as long as a day [17].

**Figure 1:**
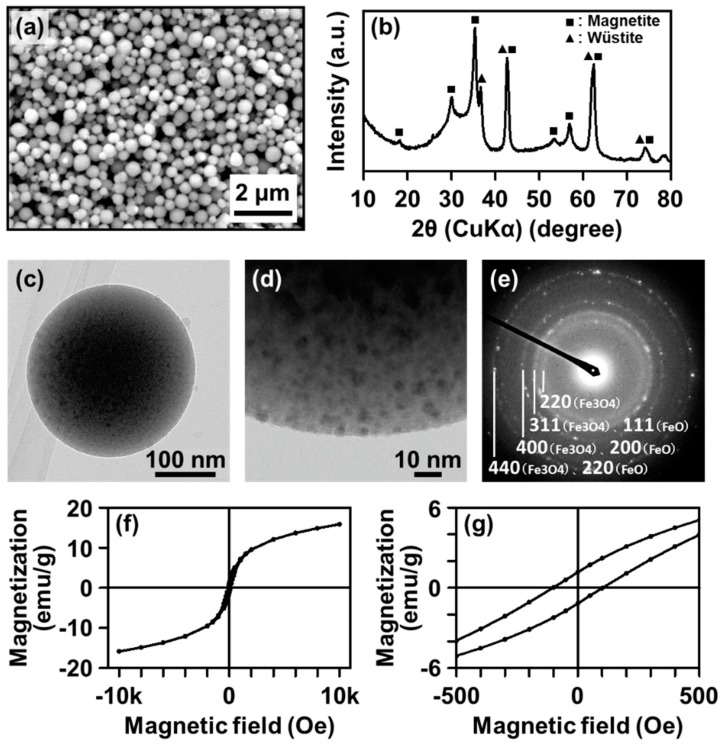
(**a**) SEM image, (**b**) XRD pattern, (**c**,**d**) TEM images (whole picture (**c**) and outer region (**d**)), (**e**) TED image, and (**f**,**g**) magnetization curves at 300 K ((**g**) is the magnified curve around the origin) of the IO–CaP submicrospheres (reproduced from [18] with permission from the PCCP Owner Societies).

We recently developed a facile one-pot fabrication process of CaP-based submicrospheres containing IO nanoparticles (denoted by IO–CaP submicrospheres) using a laser-assisted precipitation process (Figure 1a) [18,19]. In this process, weak pulsed laser irradiation was applied to a pH-controlled labile CaP reaction mixture (a mixture of calcium and phosphate ion solutions) supplemented with ferrous ions (Fe^2+^). Under laser irradiation, IO–CaP submicrospheres formed spontaneously within only 20 min in one pot. Thus, this process is advantageous in simplicity and processing time over conventional processes. The X-ray diffraction (XRD) and transmission electron diffraction (TED) analysis indicated that the resulting submicrospheres contained amorphous CaP (or amorphous CaP-based materials) along with IO crystals (Figure 1b,e). Preliminary analysis by transmission electron microscopy (TEM) revealed the presence of higher density nanoparticles dispersed in the lower density matrix of the IO–CaP submicrosphere (Figure 1c,d). In the magnetization curve (Figure 1f,g), a finite coercive field (*H*_c_ ~ 100 Oe) indicated the existence of a magnetic component in the submicrospheres that showed their potential for various biomedical applications as described earlier.

**Figure 2:**
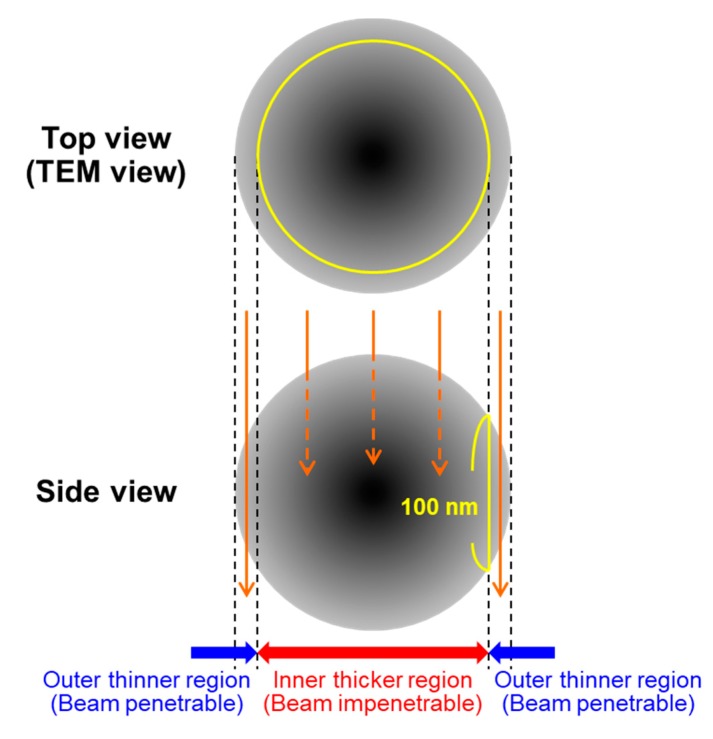
Schematic illustration of the outer thinner region and the inner thicker region of the submicrosphere.

The remaining issue in this fabrication process is the elucidation of the whole internal structure of the IO–CaP submicrospheres. We considered that the lower density matrix of the IO–CaP submicrospheres in Figure 1d should be CaP (or a CaP-based material) and the dispersed higher density nanoparticles should be IO; however, no direct evidence could support this hypothesis. In addition, our preliminary TEM analysis revealed the structure of only an outer thinner region (<100 nm) of the submicrospheres because the inner thicker region (>100 nm) is impenetrable to electron beams (Figure 2) [20]. In other words, the whole structure of the IO–CaP submicrospheres (e.g., the size and distribution of IO crystals in the submicrospheres) is still unclear.

The whole internal structure of the IO–CaP submicrospheres may provide new insight into the formation mechanism of IO–CaP submicrospheres in this fabrication process. The putative formation mechanism of the IO–CaP submicrospheres is proposed as follows, based on the hypothesis that they are composed of an amorphous CaP-based matrix and dispersed IO nanoparticles [18]. Upon preparation of the CaP reaction mixture, it promptly induces homogeneous CaP nucleation and forms irregularly-shaped iron-containing CaP precipitates. During the pulse duration, the precipitates dispersed in the reaction mixture absorb laser light, and as a result, they are heated and melted into spherical droplets. During the subsequent interval between the laser pulses, the melted droplets are quenched and solidified in an aqueous solvent, and their spherical shapes are retained. In parallel, the ferrous ions added to the reaction mixture are partially oxidized to ferric ions (Fe^3+^) and form IO nanoparticles within the CaP-based matrix.

The aim of this study was to reveal the whole internal structure of the IO–CaP submicrospheres. These aspects are essential for further development of this fabrication process and applications of the resulting materials. Herein, we performed an in-depth TEM analysis of the submicrospheres by preparing their cross-sectional specimens using resin embedding followed by argon ion milling. The formation mechanism of the IO–CaP submicrospheres was discussed on the basis of their whole structure.

## 2. Materials and Methods

### 2.1. Preparation of the IO–CaP Submicrospheres

We prepared IO–CaP submicrospheres as described in our previous paper [18]. First, aqueous CaCl_2_ (Nacalai Tesque, Inc., Kyoto, Japan; 200 mM), aqueous K_2_HPO_4_∙3H_2_O (Nacalai Tesque, Inc., Kyoto, Japan; 200 mM), and aqueous FeCl_2_∙4H_2_O (FUJIFILM Wako Pure Chemical Corporation, Osaka, Japan; 200 mM) were prepared as calcium, phosphate, and ferrous ion source solutions, respectively. A reaction mixture (4 mL) was prepared by mixing these three ion source solutions and 1 M NaOH solution (FUJIFILM Wako Pure Chemical Corporation, Osaka, Japan). Nominal concentrations of Ca, P, Fe, and NaOH in the reaction mixture were 16.7 mM, 10.0 mM, 20.0 mM, and 25.0 mM, respectively. To this reaction mixture while stirring, pulsed laser irradiation (30 Hz, 355 nm, 200 mJ/pulse/cm2) without focusing was immediately performed using a neodymium-doped yttrium aluminum garnet (Nd:YAG) laser (Quanta-Ray LAB-150-30, Spectra-Physics, Santa Clara, CA, USA). After irradiation for 20 min, the precipitates were collected by centrifugation (6000 rpm, 10 min), washed twice with ultrapure water, and dispersed again in ultrapure water. The precipitate-dispersed solution was centrifuged three times at 1000 rpm for 10 min to collect submicron-sized particles. The collected particles were dispersed again in ultrapure water, purified using a permanent magnet, and finally dried under reduced pressure at room temperature.

### 2.2. Cross-Sectional Analysis of the IO–CaP Submicrospheres

Cross-sectional specimens were prepared by conventional mechanical thinning followed by argon ion milling as detailed below. The dried final products were embedded in an epoxy resin and cured between two silicon substrates. They were cut into some cross-sectional slabs, mechanically thinned to ~100 μm, and then dimpled to ~30 μm in the center of the specimen. Final thinning was performed by argon ion milling in a precision ion polishing system (PIPS; Model 691, Gatan, Pleasanton, CA, USA). The incident beam angle and voltage were ±3° and 2.0–5.5 kV, respectively. A specimen cooling system with a liquid nitrogen stage was used during ion milling.

The prepared cross-sectional ultra-thin samples were analyzed using an analytical TEM system (Tecnai Osiris, FEI, Hillsboro, OR, USA) operated at 200 kV equipped with an energy dispersive X-ray spectrometer (EDX; Super-X system, FEI, Hillsboro, OR, USA) and a high-angle annular dark-field scanning TEM (HAADF-STEM) system with a probe diameter of ~1 nm. A total of 71 IO–CaP submicrospheres were analyzed for their internal structures. Crystalline structures of the IO–CaP submicrospheres were examined by selected area electron diffraction (SAED) and high-resolution TEM (HRTEM) analyses.

## 3. Results

### 3.1. TEM and HAADF-STEM Observation

**Figure 3:**
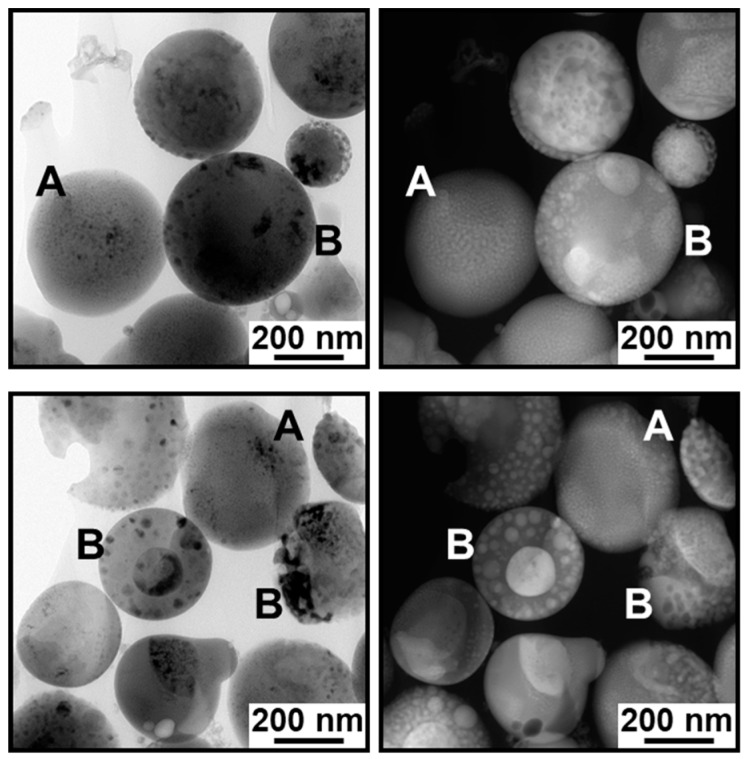
TEM (**left**) and HAADF-STEM (**right**) images of the representative cross-sectional specimens of the IO–CaP submicrospheres. A: Type A submicrospheres containing single nano-sized (<10 nm) higher density nanoparticles homogeneously dispersed throughout the lower density matrix. B: Type B submicrospheres containing larger higher density nanoparticles, which were mostly a few tens of nanometers (10–100 nm) in size along with one or two submicron-sized (>100 nm) domains.

**Figure 4:**
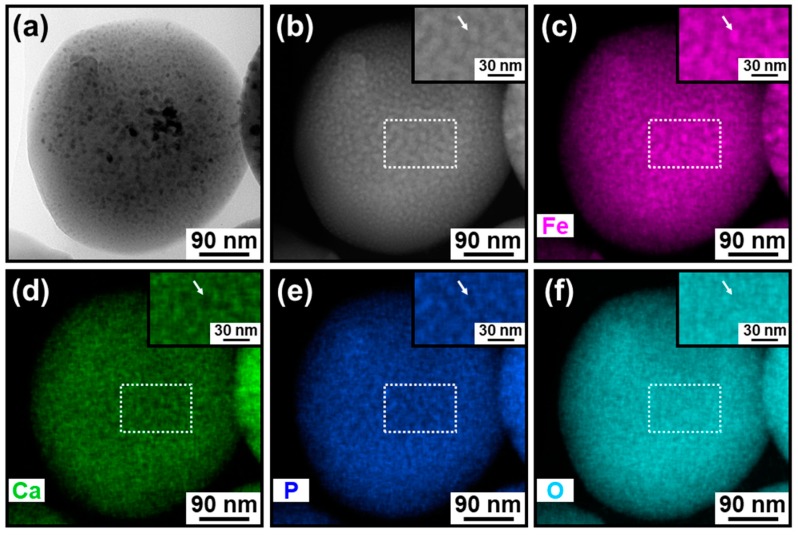
(**a**) TEM and (**b**) HAADF-STEM images and the elemental distributions of (**c**) iron, (**d**) calcium, (**e**) phosphorus, and (**f**) oxygen of the cross-sectional specimen of the type A IO–CaP submicrosphere. Inset: magnified images of the white rectangular areas (dotted line).

**Figure 5:**
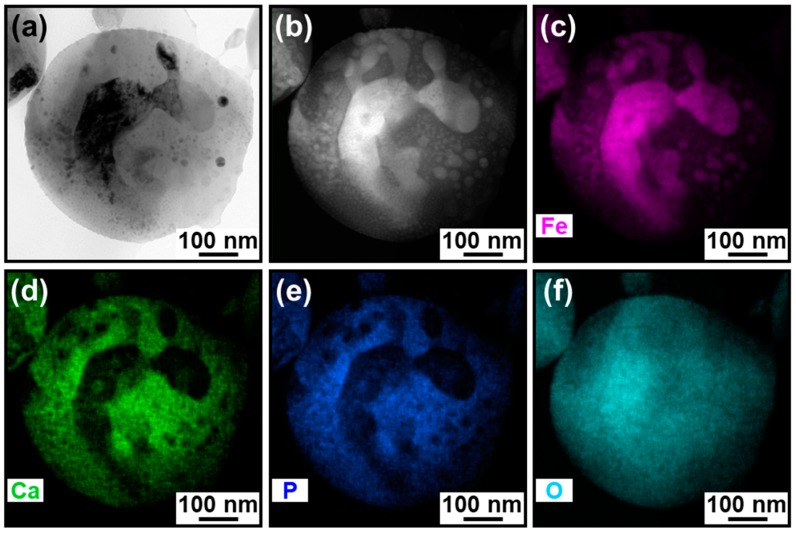
(**a**) TEM and (**b**) HAADF-STEM images and the elemental distributions of (**c**) iron, (**d**) calcium, (**e**) phosphorus, and (**f**) oxygen of the cross-sectional specimen of the type B IO–CaP submicrosphere containing irregularly-shaped IO nanoparticles.

**Figure 6:**
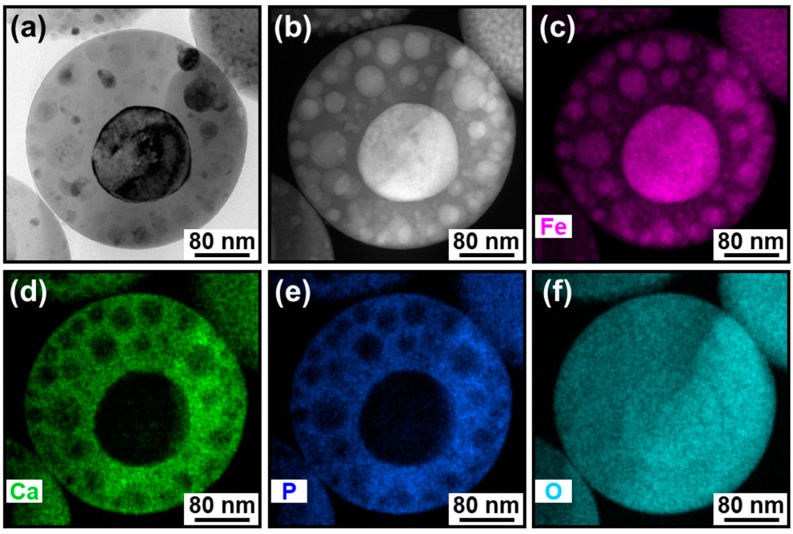
(**a**) TEM and (**b**) HAADF-STEM images and the elemental distributions of (**c**) iron, (**d**) calcium, (**e**) phosphorus, and (**f**) oxygen of the cross-sectional specimen of the type B IO–CaP submicrosphere containing spherical IO nanoparticles.

The cross-sectional TEM analysis revealed that the IO–CaP submicrospheres had variations in their nanostructures. Figure 3 shows the TEM (left) and HAADF-STEM (right) images of the representative two cross-sectional specimens. Higher density nanoparticles were found not only in the outer region but also in the inner region of the submicrospheres, as indicated by the darker spots in the TEM images (Figure 3, left) and the brighter spots in the HAADF-STEM images (Figure 3, right). The observation results indicate that the submicrospheres were roughly divided into two types (i.e., types A and B) depending on the size, shape, and distribution of the higher density nanoparticles in the submicrospheres. The type A submicrospheres (reported in our previous study [18]) contained single nano-sized higher density nanoparticles homogeneously dispersed throughout the lower density matrix (Figure 4a,b). The type B submicrospheres contained higher density nanoparticles, which were mostly a few tens of nanometers (10–100 nm) in size, dispersed throughout the lower density matrix along with one or two larger domains (>100 nm) (Figure 5a,b and Figure 6a,b). The existence ratio of the type A submicrospheres and type B submicrospheres was approximately 3:7 (estimated from a total of 71 submicrospheres in 16 TEM/HAADF-STEM images). The type B submicrospheres were further divided into two types depending on the shape of the higher density nanoparticles. A large majority (ca. 90%) of the type B IO–CaP submicrospheres had irregularly-shaped higher density nanoparticles (both the smaller and larger ones) as depicted in Figure 5a,b. In addition to this type of submicrospheres, we observed type B IO–CaP submicrospheres containing spherical higher density nanoparticles (Figure 6a,b), but very occasionally (ca. 10%). For both type A and type B, the higher density nanoparticles were embedded within the lower density matrix of the submicrosphere and not located on its outside surface. The type B IO–CaP submicrospheres were newly found in this study.

### 3.2. Elemental Analysis

For both types of IO–CaP submicrospheres, the lower density matrix and the dispersed higher density nanoparticles were CaP (or a CaP-based material) and IO, respectively, as previously predicted [18]. The elemental distributions were investigated by STEM-EDX for each type of the submicrospheres (types A and B) (Figure 4c–f, Figure 5c–f and Figure 6c–f). In the type A submicrospheres, distribution of the single nano-sized higher density nanoparticles indicated by the brighter spots in the HAADF-STEM image (Figure 4b) corresponded to the iron distribution (Figure 4c, see the magnified image in the inset). Both calcium (Figure 4d) and phosphorus (Figure 4e) showed complementary distributions to the iron distribution (Figure 4c). Similar STEM-EDX results were also obtained for the type B submicrospheres (Figure 5 and Figure 6). The lower density matrix was rich in calcium (Figure 5d and Figure 6d) and phosphorus (Figure 5e and Figure 6e), while the dispersed higher density nanoparticles were rich in iron (Figure 5c and Figure 6c). These results clarified the whole structure of the IO–CaP submicrospheres. They were composed of a CaP-based matrix with dispersed IO nanoparticles for both types of submicrospheres (types A and B).

### 3.3. Electron Diffraction Analysis

**Figure 7:**
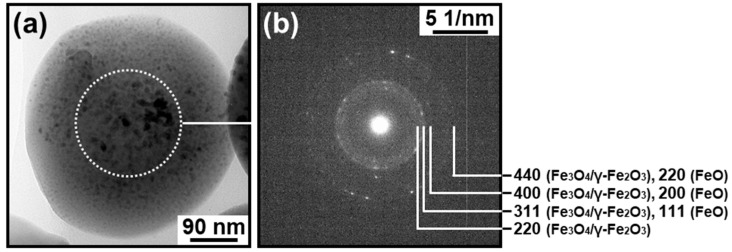
(**a**) TEM image and (**b**) SAED pattern taken from the white circular area (dotted line) in (**a**) of the cross-sectional specimen of the type A IO–CaP submicrosphere.

**Figure 8:**
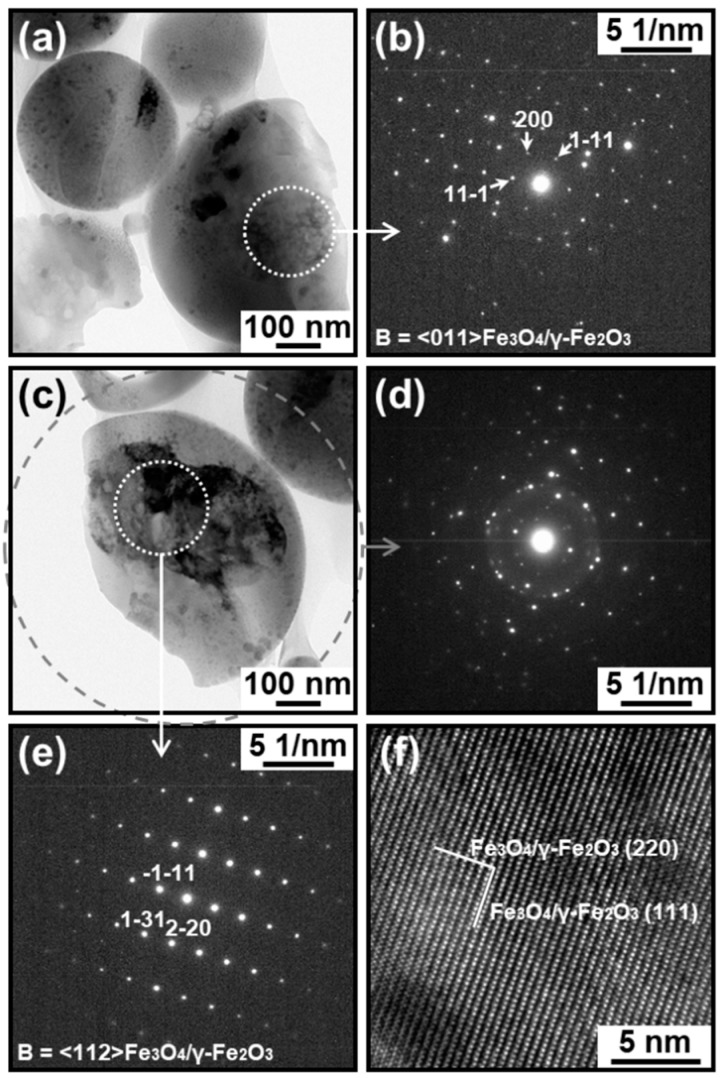
(**a**,**c**) TEM images, (**b**,**d**,**e**) SAED patterns, and (**f**) HRTEM image of the cross-sectional specimens of the type B IO–CaP submicrospheres. The SAED patterns in (**b**,**d**,**e**) are taken from the white circular area (dotted line) in (**a**), the gray circular area (broken line) in (**c**), and the white circular area (dotted line) in (**c**), respectively. The HRTEM image is obtained from the white circular area (dotted line) in (**c**).

The presence of crystalline IO in the IO–CaP submicrospheres was reconfirmed by electron diffraction analysis. The inner region of the type A submicrosphere showed an SAED pattern ascribed to magnetite (Fe_3_O_4_), maghemite (γ-Fe_2_O_3_), and wüstite (FeO) (Figure 7b). No apparent diffraction spot or ring was attributable to crystalline CaPs in the SAED pattern and, therefore, suggested that the CaP-based matrix was amorphous. The type B submicrosphere also showed an SAED pattern ascribed to magnetite and maghemite with a cubic space group *Fd3m* (possibly coexisting with wüstite [18]) (Figure 8b,d). Note that there are three possible space groups for maghemite: a cubic space group *Fd3m* (same as magnetite), a cubic space group *P4_1_32*, and a tetragonal space group *P4_3_2_1_2* [21]. In the large domain (>100 nm) in the inner region of this submicrosphere (the white circular area in Figure 8c), an SAED pattern corresponding to monocrystalline magnetite and maghemite (*Fd3m*) was obtained (Figure 8e). Additionally, in the HRTEM image of this region, lattice patterns indexed to the (111) and (220) planes of magnetite and maghemite were observed (Figure 8f). We observed similar SAED results for several other submicrospheres of type A and type B. These results and our previous XRD results suggesting the coexistence of wüstite as a minor phase (Figure 1b) [18] clarified that the dispersed nanoparticles were crystalline IO: magnetite and/or maghemite along with a small amount of wüstite.

## 4. Discussion

The cross-sectional TEM analysis of the IO–CaP submicrospheres fabricated by our laser-assisted precipitation process [18] revealed that they were composed of an amorphous CaP-based matrix and dispersed IO (magnetite and/or maghemite, and wüstite) nanoparticles with various sizes and shapes. As hypothesized in our previous study, the IO nanoparticles were distributed throughout the CaP-based matrix of the submicrosphere. In other words, they existed not only in the outer region, but also within the inner region of the submicrosphere. However, the IO–CaP submicrospheres were inhomogeneous in their internal nanostructures (i.e., size, shape, and distribution of the IO nanoparticles). The type A IO–CaP submicrosphere contained single nano-sized IO nanoparticles that were densely and homogeneously dispersed throughout the CaP-based matrix (Figure 4), which corresponded to the structure expected in our previous study [18]. In addition to this type A submicrosphere, a different type (type B) of the IO–CaP submicrosphere was newly found. It contained nanoparticles, which were mostly a few tens of nanometers (10–100 nm) in size along with one or two larger domains (>100 nm) (Figure 5 and Figure 6).

Considering the whole structure of the IO–CaP submicrospheres described earlier, their formation mechanism can be described in the following manner. The reaction mixture containing calcium, phosphate, and ferrous ions induces oxidization of some ferrous ions to ferric ions while inducing homogeneous CaP nucleation (amorphous phase nucleation). The resulting ferrous–ferric coexistence condition is favorable for precipitation of magnetite and maghemite leading to the formation of CaP-based precipitates containing magnetite and/or maghemite (possibly coexisting with wüstite) in the reaction mixture. These reactions proceed even without laser irradiation, albeit very slowly as confirmed by the control experiment without laser irradiation [18]. Pulsed laser light irradiation accelerates IO crystallization in the CaP-based precipitates by selectively heating the precipitates in the liquid phase. The selective heating is because the iron-containing precipitates absorb laser light energy, while the surrounding liquid phase hardly dose so. The initially formed IO nanoparticles have the size of single nanometers and are dispersed throughout the CaP-based matrix. These IO nanoparticles and the CaP-based matrix momentary melt and spheroidize during the pulse duration. The thus-formed IO–CaP submicrospheres are categorized as type A. In some submicrospheres, single nano-sized IO nanoparticles fuse together and become larger (10–100 nm) in the momentary molten state under laser irradiation. Some of these IO nanoparticles further aggregate and merge to form a larger domain (>100 nm) in the submicrosphere. The thus-formed IO–CaP submicrospheres are categorized as type B. In most of the type B submicrospheres, the merged IO nanoparticles had an irregular shape (Figure 5), whereas they had a spherical shape in some rare cases (Figure 6). The mechanism causing this difference is yet to be clarified. However, we consider that the latter type B submicrospheres (rare case) might be formed from the former type B submicrospheres through melting and spheroidizing of the merged IO nanoparticles within the CaP-based matrix during pulse duration, followed by their solidification with retaining their spherical shapes during pulse interval. Our previous XRD analysis [18] suggested the coexistence of a small amount of wüstite in the submicrospheres although wüstite was not clearly detected in either type of submicrospheres by the present SAED analysis because of the interference with magnetite and maghemite. Wüstite might be formed from the preformed magnetite (or maghemite) nanoparticles. Magnetite/wüstite composite submicrospheres were prepared from pure magnetite nanoparticles by pulsed laser irradiation in a liquid [22,23]. A similar phenomenon (i.e., the partial transformation of magnetite into wüstite) might also occur in the CaP-based submicrospheres in the present laser irradiation process.

As described in the Introduction section, IO–CaP submicrospheres have considerable potential in various biomedical applications. Our laser-assisted precipitation process is simple (one-pot) and rapid (short-time irradiation), and requires no surfactants. Therefore, it is advantageous over conventional fabrication processes of similar IO–CaP composite particles [14,15,16,17]. However, the IO–CaP submicrospheres fabricated by our process were inhomogeneous in their nanostructures (categorized as type A and type B). These mixed submicrospheres should be purified before use or the process parameters (e.g., laser fluence, irradiation time, and reaction mixture composition) should be adjusted to produce homogeneous IO–CaP submicrospheres because the optimum structures of IO nanoparticles are different among their application purposes [4,5,6]. In this regard, analysis of the whole structure of the IO–CaP submicrospheres presented herein is inevitable for the further development of this fabrication technique and the resulting IO–CaP submicrospheres.

## 5. Conclusions

In this study, the cross-sectional TEM analysis clarified the whole structure of the IO–CaP submicrospheres fabricated by our laser-assisted precipitation process. The IO–CaP submicrospheres were composed of an amorphous CaP-based matrix and dispersed IO nanoparticles with various sizes and shapes. Depending on their internal nanostructures, the IO–CaP submicrospheres were categorized into two types (i.e., A and B). The type A submicrospheres contained many single nano-sized IO nanoparticles homogeneously dispersed throughout the CaP-based matrix. The type B submicrospheres contained larger IO nanoparticles, which were mostly a few tens of nanometers in size along with one or two submicron-sized domains. These findings provided new insight into the formation mechanism of the IO–CaP submicrospheres in the present process. For future applications of these IO–CaP submicrospheres, purification of type A/B submicrospheres, or further process refinement to produce homogeneous IO–CaP submicrospheres is required.
